# Fabrication of Multi-Vacancy-Defect MWCNTs by the Removal of Metal Oxide Nanoparticles

**DOI:** 10.3390/polym14142942

**Published:** 2022-07-20

**Authors:** Tae Hyeong Kim, Dong Hwan Nam, Do-Hyun Kim, Gyu Leem, Seunghyun Lee

**Affiliations:** 1 Department of Applied Chemistry, Hanyang University ERICA, Ansan 15588, Korea; thkim1231@hanyang.ac.kr (T.H.K.); dhnam12@hanyang.ac.kr (D.H.N.); 2 Center for Bionano Intelligence Education and Research, Hanyang University ERICA, Ansan 15588, Korea; 3 School of Electrical Engineering, Korea University, Seoul 02841, Korea; nanotube@korea.ac.kr; 4 Department of Chemistry, State University of New York, College of Environmental Science and Forestry, 1 Forestry Drive, Syracuse, NY 13210, USA; gyleem@esf.edu; 5 The Michael M. Szwarc Polymer Research Institute, 1 Forestry Drive, Syracuse, NY 13210, USA; 6 Department of Chemical and Molecular Engineering, Hanyang University ERICA, Ansan 15588, Korea

**Keywords:** multi-walled carbon nanotubes, metal oxide NPs, multi-vacancy-defect

## Abstract

This study aims to increase the specific surface area of multi-walled carbon nanotubes (MWCNTs) by forming and subsequently removing various metal oxide nanoparticles on them. We used facile methods, such as forming the particles without using a vacuum or gas and removing these particles through simple acid treatment. The shapes of the composite structures on which the metal oxide particles were formed and the formation of multi-vacancy-defect MWCNTs were confirmed via transmission electron microscopy and scanning electron microscopy. The crystallinity of the formed metal oxide particles was confirmed using X-ray diffraction analysis. Through specific surface area analysis and Raman spectroscopy, the number of defects formed and the degree and tendency of defect-formation in each metal were determined. In all the cases where the metal oxide particles were removed, the specific surface area increased, and the metal inducing the highest specific surface area was determined.

## 1. Introduction

Carbon nanotubes (CNTs) with excellent mechanical, electrical, thermal, and chemical properties [1,2,3] are novel materials for diverse future applications and are expected to be widely employed in various industrial applications in the long term. Conventionally, metallic and ceramic materials have been employed in various applications, such as those requiring conductive and high-strength support materials. However, these materials possess disadvantages, such as heavy weight and brittleness. Carbon-based materials have recently attracted attention as promising alternatives to these conventional materials. Carbon exhibits various allotropic forms, such as graphite, diamond, fullerene, CNTs, carbon fibers, and graphene [4,5]. CNTs were discovered by Dr. S. Iijima in 1991 [6]. The synthesis methods of CNTs have evolved from conventional laser vaporization [7,8] or electrical discharge methods [9,10] to chemical vapor deposition (CVD) [11,12,13]. CNTs are also used as energy electrodes owing to their higher specific surface area for the same mass as the other carbon-based materials [14,15]. CNTs are being developed as a variety of catalysts with excellent adsorption power [16,17], electron emission sources with excellent electrical characteristics for field emission displays [18,19], lithium-ion secondary battery electrodes [14,20,21,22,23], and as probes for atomic force microscopy [24]. In addition, ultrasmall and ultrathin electromagnetic shielding films and heating elements [25,26,27,28] based on CNTs are being industrialized. Additionally, by utilizing the excellent mechanical properties of CNTs, it is also used as a material for nanocomposite used as an electrochemical sensor [29].

CNTs have a very high specific surface area owing to their diameters in the nanometer range [30]. In particular, CNTs are promising materials for hydrogen storage, owing to their physical adsorption property of van der Waals interactions. CNTs are stored in internal channels or between individual CNTs while retaining their nanostructures and high specific surface area characteristics [31,32,33,34]. CNTs also have excellent adsorption capacities because of their π–π electron interactions and hydrophobic interactions on the surface [35,36,37,38]. Owing to such adsorption characteristics, they can be used as adsorbents for separating pollutants from wastewater or as a support for catalysts for adsorbing organic pollutants [39,40]. In an effort to improve their characteristics, CNTs have been grown with a high specific surface area [41] or with defects [42]. However, increasing their specific surface area using various metals has not yet been reported. The resilience of multi-walled carbon nanotubes (MWCNTs) increases after forming defects in ion-irradiation [43]. It has also been reported that even minor defects formed via heating and ultrasonic treatment participate in catalytic activity and provide stability. Dangling bonds due to the defects also play an important role in the catalytic reaction because they facilitate contact with important materials in applications [44,45].

**Figure 1:**
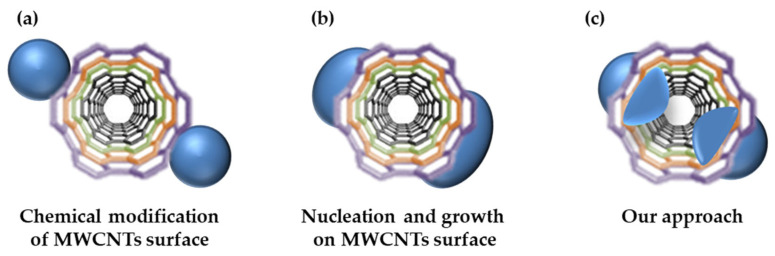
Different approaches for the fabrication of MWCNT@metal oxide NP composites.

Herein, we report a new approach for the formation of particles on the surface of CNTs. The conventional MWCNT-metal oxide composite fabrication is shown in Figure 1a,b. In Figure 1a, the bonding between CNTs and metal oxide is chemical/electrostatic bonding that is achieved by modifying the surface of the as-fabricated nanoparticles (NPs) and CNTs. Because the NPs were simply attached to the CNT surface, they formed a composite while maintaining their original shape. Figure 1b shows the formation of metal NPs on the surface of the modified CNTs via nucleation and growth. However, in this case, the metal NPs can overgrow and cover the CNTs, which is disadvantageous. Moreover, in the aforementioned methods, the NPs are formed only on the outer walls of the CNTs and could not penetrate the CNT walls. However, in this study, (Figure 1c), NPs were grown inward from the outermost wall of the MWCNTs, so that they could penetrate the CNT walls. The subsequent removal of NPs increased the specific surface area, and the formation of dangling bonds also increased the adsorption capacity. Because of the use of metals is more diverse than in previous composite fabrication studies, this study can identify the particle formation tendency for each metal [46]. In addition, it may be used in different application parts for each composite of metal oxides, and multi-vacancy-defect MWCNTs may also be applied as a catalyst such as organic pollutants degradation [47]. The formation of the NPs and defects was confirmed using transmission electron microscopy (TEM), and the crystallinity of the NPs was determined using X-ray diffraction (XRD) patterns. Through Brunauer–Emmett–Teller (BET) surface area analysis and G/D ratio analysis via Raman spectroscopy, it was confirmed that the NPs were successfully removed, and the specific surface area increased. Additionally, the precursor metal that yields the highest specific surface area (maximum defects) was determined.

## 2. Materials and Methods

### 2.1. Materials

MWCNTs (JENO20) were procured from JEIO (Yusong, Korea). For the purification process, which involves the removal of amorphous carbon present on the surface of the CNTs and the formation of an oxygen functional group, sulfuric acid (98%) and nitric acid (60%) were procured from DAEJUNG Chemicals (Siheung-si, Korea). For the formation of transition metal oxides on the surface of CNTs, cobalt (II) nitrate hexahydrate, iron (III) nitrate nonahydrate, manganese (II) nitrate hexahydrate, and nickel (II) nitrate hexahydrate were procured from Sigma-Aldrich (Burlington, MA, USA) and used as transition metal hydrates. Deionized water (DIW) was used to dissolve the transition-metal hydrates. Ethyl alcohol (99.9%), procured from DUKSAN (Ansan, Korea), was used as a solvent to disperse the CNTs, and ammonium hydroxide was procured from JUNSEI (Tokyo, Japan).

### 2.2. Purification of CNTs

**Figure 2:**
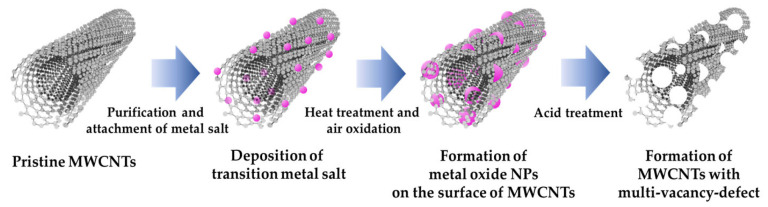
Schematic diagram of the entire experimental process.

Figure 2 shows schematic diagram of our experiment. Prior to the purification, the CNTs were heat-treated to remove amorphous carbon, for which 1 g of MWCNTs were placed in a crucible and heat-treated in a muffle furnace at 450 °C for 2 h. After removing the amorphous carbon, 1 g of MWCNTs were added to a round-bottom flask, and 40 mL of nitric acid, 5 mL of sulfuric acid, and 35 mL of DIW were subsequently added. A feedback controller was used to maintain the temperature at 40 °C and the mixture was stirred for 48 h. Subsequently, to prevent the loss of solvent vapor due to evaporation, a reflux condenser was connected, and water temperature was set to 10 °C. The purification process was conducted to obtain only the pure CNTs and attach a functional group to the surface of the CNTs. Although approximately 10% of oxygen functional groups (carboxyl, hydroxyl, and epoxy groups) are attached to the surface, it is necessary to attach as much transition metal hydrates as possible [48]. After purification, the solution was diluted to 300 mL using DIW for filtration. The solution was filtered under reduced pressure conditions using a quantitative filter paper and dried in an oven at 70 °C for 24 h.

### 2.3. Formation of Metal Oxide NPs

In a 250 mL beaker, 5 mL DIW and 0.2 g transition metal hydrate salt were sonicated for 30 min. Subsequently, 0.3 g of purified MWCNTs were added to 90 mL of ethanol and 5 mL of aqueous ammonia and were dispersed via probe sonication for 30 min. To attach the transition metal hydrate salt to the CNT surface, the solution was maintained at 78–80 °C using a heating plate and stirred to evaporate the solvents completely, followed by drying at 70 °C for 24 h. The CNTs were then placed with transition metal hydrate salt on a quartz boat and were heat-treated at 300 °C for 2 h with both gates open, using a CVD system. After the heat treatment, the samples were washed several times with distilled water, filtered under reduced pressure conditions, and dried at 70 °C for 24 h.

### 2.4. Fabrication of Porous Carbon Nanotubes by Removal of Metal Oxide NPs

After adding 0.3 g CNTs containing the as-formed NPs in a 250 mL beaker, 35 mL sulfuric acid and 5 mL of DIW were added, and the mixture was stirred for 4 h. After diluting it with 400 mL of DIW, it was filtered under reduced pressure conditions using a polytetrafluoroethylene (PTFE) membrane filter, washed several times, and dried in an oven at 70 °C for 24 h.

### 2.5. Characterization

High-resolution TEM (FEI Tecnai G2 F20 model, Hillsboro, OR, USA) imaging was used to check whether any transition metal oxides were formed up to the inner walls of the MWCNTs. FE-SEM (Hitachi S-4800, Tokyo, Japan) with energy dispersive X-ray spectroscopy (EDS) was used to check elements and content of the composite. XRD analysis was performed to determine the characteristics of the NPs identified in the TEM images of the MWCNT@metal oxide NP composites using diffractometer (Rigaku Miniflex600, Tokyo, Japan). To confirm the decrease in metal element content, X-ray photoelectron spectroscopy (XPS) (Thermo Fisher Scientific K-Alpha plus, Waltham, MA, USA) analysis was performed. The specific surface area of the composites was measured by a surface area analyzer (BEL Inc. BELSORP-mini II, Osaka, Japan) using the Brunauer–Emmett–Teller (BET) and Barrett–Joyner–Halenda (BJH) methods. Raman spectrometer (HEDA, NOST) was used to obtain and compare the G/D ratios of composites. Raman spectroscopy is a nondestructive characterization technique that uses light scattering to obtain information on the surface states and molecular structures of CNTs. This technique involves the phenomenon in which the light incident on a molecule is scattered, and phonons are emitted as vibrational energy [49].

## 3. Results and Discussion

### 3.1. TEM

**Figure 3:**
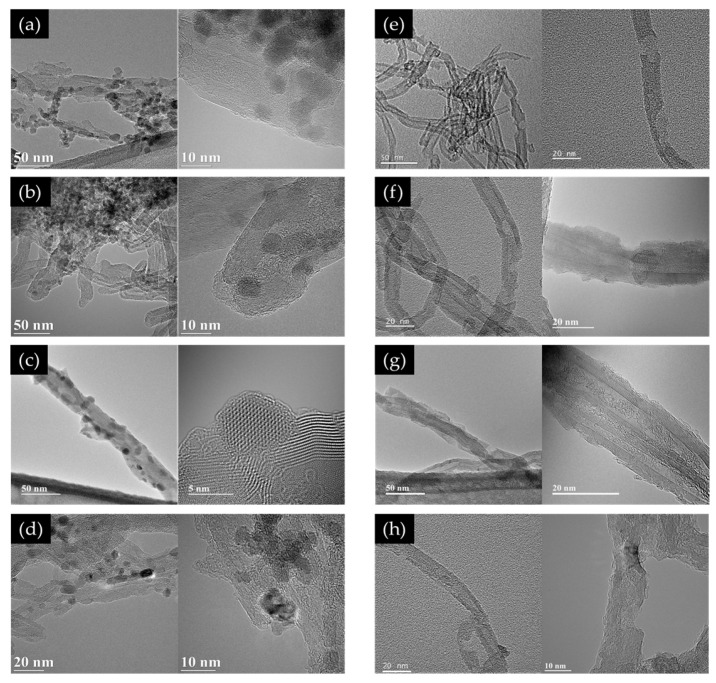
TEM images of (**a**–**d**): MWCNT@metal oxide NP composites; (**e**–**h**): multi-vacancy-defect MWCNTs after the removal of metal oxide NPs.

MWCNT@metal oxide NP composites were prepared using Co, Fe, Mn, and Ni as transition metal precursors. TEM observations confirm the formation of metal oxide particles and MWCNT@metal oxide NP composites, as shown in Figure 3a–d. Thereafter, the metal oxide NPs were removed to form multi-vacancy-defect MWCNTs, which were also analyzed using TEM, and the images are shown in Figure 3e–h. Unlike the purified MWCNTs, defects were found in all composites from which all the transition metal NPs were removed.

### 3.2. SEM/EDS

**Figure 4:**
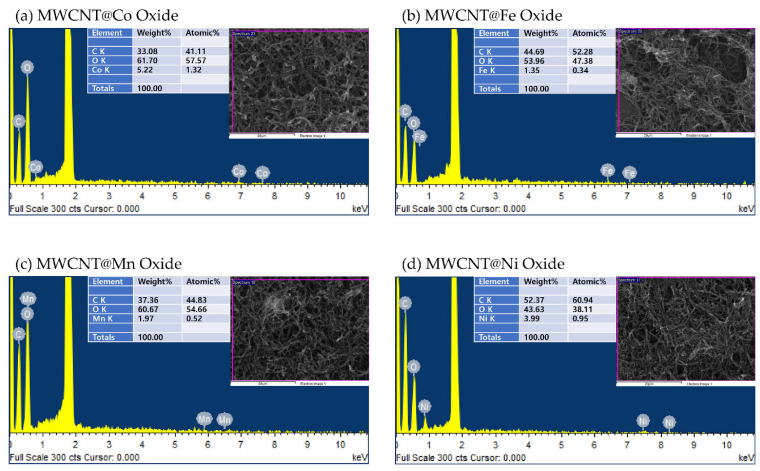
SEM images and EDS spectrum with content of MWCNT@metal oxide NP composites.

SEM/EDS observations also confirm the formation of metal oxide particles and MWCNT@metal oxide NP composites, as shown in Figure 4. As a result of confirming the SEM image, the formation of each metal oxide particles on the MWCNTs was confirmed. In addition, the presence and content of each element were checked using EDS. As a result, the elements of the metal used in each composite were measured, and cobalt showed the highest content.

### 3.3. XRD

**Figure 5:**
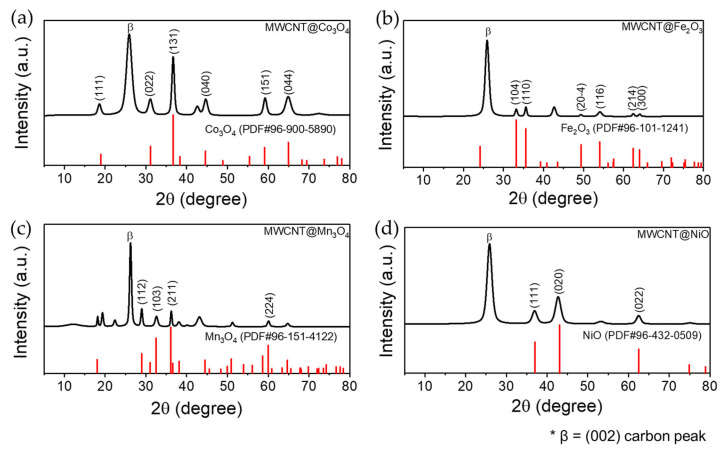
XRD patterns for the CNT composites (**a**) MWCNT@Co_3_O_4_, (**b**) MWCNT@Fe_2_O_3_, (**c**) MWCNT@Mn_3_O_4,_ and (**d**) MWCNT@NiO. The beta mark is a common peak for carbon (002).

The diffraction spectra of the composites are shown in Figure 5. The peaks at approximately 26° and 44° are attributed to MWCNTs and correspond to (003) and (101) crystal planes, respectively. Figure 5a shows the XRD spectrum of the MWCNT@Co oxide NP composite. The peaks at 18°, 31°, 36°, 44°, 59°, and 64° correspond to the (111), (022), (131), (040), (151), and (044) crystal planes of cubic Co_3_O_4_ (PDF#96-900-5890), respectively, and therefore, the produced NPs could be cobalt oxide. The peak at 44° appears to be an overlap of two peaks. Considering the spectrum of the MWCNT@Fe oxide NP composite (Figure 5b), the peaks at 33°, 35°, 49°, 54°, 62°, and 63° correspond to the (104), (110), (20-4), (116), (214), and (300) planes of hexagonal Fe_2_O_3_ (PDF#96-101-1241). Therefore, the NPs formed were Fe_2_O_3_. From the XRD spectrum of the MWCNT@Mn oxide NP composite (Figure 5c), the peaks at 28°, 32°, 36°, and 60° correspond to the (112), (103), (211), and (224) planes of tetragonal Mn_3_O_4_ (PDF#96-151-4122), and the NPs produced could be manganese oxide. Finally, the peaks in the spectrum of the MWCNT@Ni oxide NP composite (Figure 5d) at 37°, 43°, and 62° correspond to the (111), (020), and (022) planes of cubic NiO (PDF#96-432-0509). Therefore, it was concluded that the produced NPs were NiO. The NiO peak also appears to overlap with the (101) peak (at 44°) of the MWCNTs, as in the case of the cobalt composite. XRD analysis thus confirmed the formation of metal oxide (Co_3_O_4_, Fe_2_O_3_, Mn_3_O_4_, and NiO) NPs on the MWCNTs.

### 3.4. XPS

**Figure 6:**
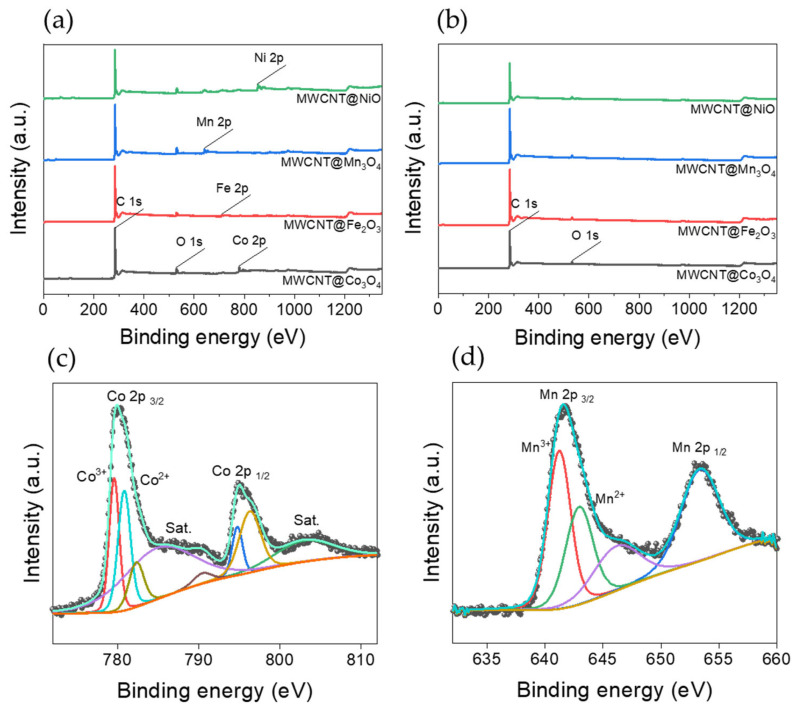
XPS survey spectra of (**a**) MWCNT@metal oxide NP composites, (**b**) multi-vacancy-defect MWCNTs and deconvoluted high-resolution XPS spectra of (**c**) Co 2p of MWCNT@Co_3_O_4_, (**d**) Mn 2p of MWCNT@Mn_3_O_4_.

Figure 6a,b shows the XPS survey spectra of MWCNT@metal oxide NP composites and multi-vacancy-defect MWCNTs, respectively. This is to confirm that the particles are clearly separated, and defects are formed. The XPS survey spectra of the MWCNT@metal oxide NP composites showed the peaks of Co 2p, Fe 2p, Mn 2p, and Ni 2p elements used in each composite at binding energies of 780.08 eV, 711.08 eV, 642.08 eV, and 855.08 eV, respectively. Figure 6b shows that the peak of each metal element shown in Figure 6a decreased and most disappeared. This proves that the metal oxide NPs formed on the MWCNTs were successfully removed.

In the case of the XRD patterns of MWCNT@Co_3_O_4_ and MWCNT@Mn_3_O_4_, overlapped peaks exist in different phases of the same metal oxide. Therefore, high-resolution XPS was measured and shown in Figure 6c,d to confirm once more that NPs were Co_3_O_4_ and Mn_3_O_4_, respectively. The deconvoluted XPS spectra of Co 2p showed two main peaks at the binding energies of 780.08 eV and 794.88 eV, respectively, corresponding to 2p 3/2 and 2p 1/2 along with their corresponding satellite peaks at the binding energies of 784.82 eV and 803.00 eV, respectively, similar to those observed in Co_3_O_4_ reported previously [50]. Additionally, the deconvoluted XPS spectra of Mn 2p showed two main peaks at the binding energies of 642.08 eV and 652.88 eV, respectively, corresponding to 2p 3/2 and 2p 1/2 similar to those observed in Mn_3_O_4_ reported previously [51]. In the case of Co_3_O_4_, which is actually a mixed oxidation state of Co(II) and Co(III), expect to see peaks due to the Co^2+^ and Co^3+^ states. In addition, the case of Mn_3_O_4_ is also the same. For Co 2p, by the fitted wide main peak, it is reasonable to assume that Co^2+^ and Co^3+^ coexists, and by the fitted wide main peaks of Mn 2p, it can be assumed that Mn^2+^ and Mn^3+^ coexists. Therefore, in the case of Co and Mn NPs, it shows that 2+ and 3+ states coexist as Co_3_O_4_ and Mn_3_O_4_ as shown in the XRD results.

### 3.5. Specific Surface Area Analysis

**Figure 7:**
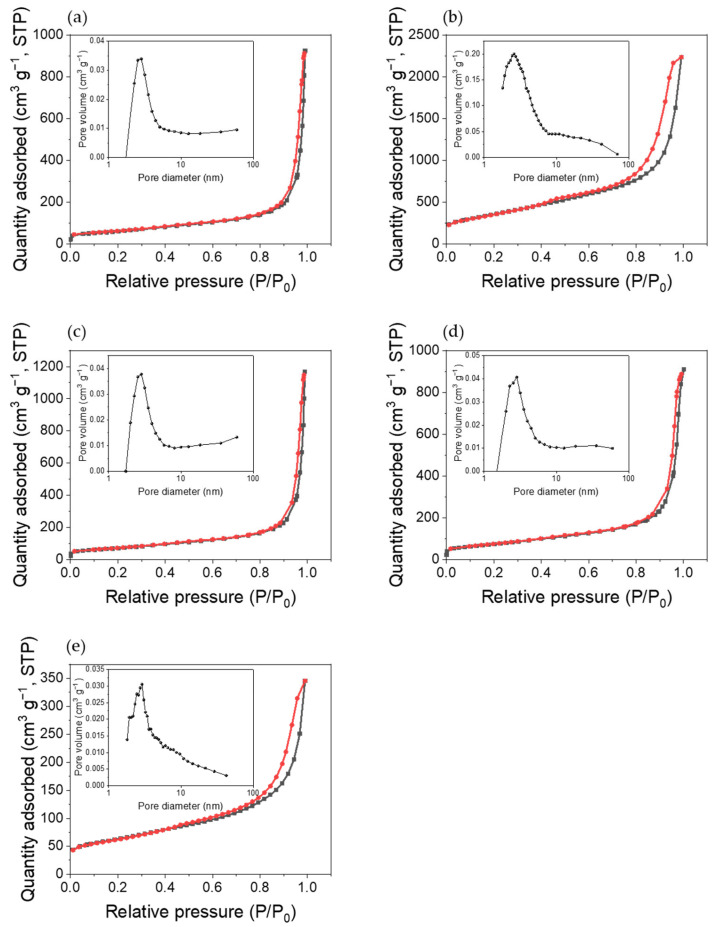
BET specific surface area and BJH pore size distribution of (**a**) purified MWCNTs and multi-vacancy-defect MWCNTs after the removal of (**b**) Co_3_O_4_, (**c**) Fe_2_O_3_, (**d**)Mn_3_O_4_, and (**e**) NiO NPs.

The specific surface areas of the purified MWCNTs and multi-vacancy-defect MWCNTs after the removal of various metal oxide NPs were measured using BET and are showed in Figure 7 and Table 1. The specific surface area of the composites increased owing to CNT defects. First, the specific surface area of the purified MWCNTs was measured as 214.01 m^2^/g, which was considered as a reference value. In the case of multi-vacancy-defect MWCNTs, the specific surface area increased after the removal of all the four metal oxides. The specific surface areas after removing Co_3_O_4_, Fe_2_O_3_, Mn_3_O_4_, and NiO were 1273.37, 253.52, 266.12, and 218.18 m^2^/g, respectively. The difference in the specific surface areas of the composites after removal of NPs from that of the purified MWCNTs was the largest for Co_3_O_4_ (1059.36 m^2^/g), followed by Mn_3_O_4_ (52.11 m^2^/g), Fe_2_O_3_ (39.51 m^2^/g), and finally, NiO (4.17 m^2^/g). Therefore, defects were generated by the removal of the metals, thereby increasing the specific surface area. Among them, the removal of Co_3_O_4_ is considered to have induced the most number of defects, as the resulting materials showed a six-fold increase in the specific surface area of purified MWCNTs at 1273.37 m^2^/g.

### 3.6. Raman Spectroscopy

**Figure 8:**
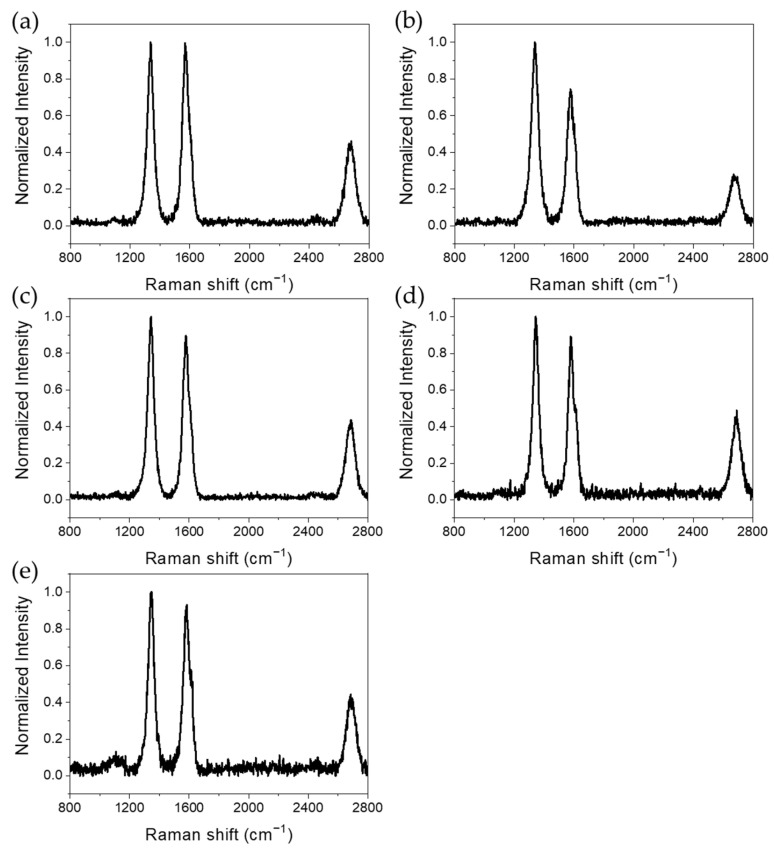
Raman spectra of (a) purified MWCNTs and multi-vacancy-defect MWCNTs after the removal of (**b**) Co_3_O_4_, (**c**) Fe_2_O_3_, (**d**) Mn_3_O_4_, and (**e**) NiO NPs.

**Figure 9:**
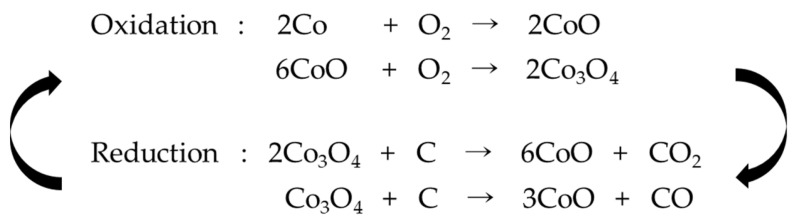
Reaction mechanism of MWCNT@Co_3_O_4_.

Raman spectroscopy was performed to obtain and compare the G/D ratios of purified MWCNTs and multi-vacancy-defect MWCNTs from which each metal oxide was removed. The G/D ratio can be used to evaluate the crystal purity and defect concentration of CNTs. The peak at approximately 1342 cm^−1^ corresponds to disordered graphite, a defect in CNTs, and the peak at approximately 1576 cm^−1^ corresponds to the E_2g_ stretching mode of graphite. These peaks correspond to the structural changes along the CNT axis [52]. Because the intensity of the D-peak in the spectrum increases with the number of defects in the CNTs, the degree of defects may be evaluated based on the G/D ratio. The intensity, which is along the *y*-axis of the spectrum, was normalized to one for easy interpretation. If the defects in the CNTs are negligible, the ratio is closer to one, and as the number of defects increases, the ratio decreases. Figure 8 and Table 2 show that the G/D ratio of the purified MWCNTs was 0.97, which is close to 1. In the case of multi-vacancy-defect MWCNTs from which each metal oxide was removed, the G/D ratio decreased, and therefore, defects in all composites were well-formed. Among them, the composite after the NiO removal showed the smallest decrease in the G/D ratio to 0.95, whereas the Co_3_O_4_ removal caused the largest decrease to 0.74. The maximum number of NPs were formed in the case of cobalt, exhibiting a trend similar to the results of the specific surface area analysis. It can be confirmed that the G/D ratio decrease trend of cobalt and other metals is the same as the specific surface area trend. Therefore, it is shown that the G/D ratio and the specific surface area are proportional. In addition, it is possible to confirm the mechanism (Figure 9) of forming Co_3_O_4_ while penetrating the outer wall of MWCNTs under oxygen conditions through previous studies [42]. Because of the repetition of this mechanism, it would have penetrated the outer wall much more than any other metals.

### 3.7. Electrical Conductivity

We confirmed that defects were formed when NPs were removed by measuring and comparing BET and BJH (Figure 7) and the G/D ratio (Figure 8). In addition, electrical conductivity was measured to confirm the multi-vacancy-defect were formed. We made thin films onto nylon membrane filters using purified MWCNTs and multi-vacancy-defect MWCNTs to acquire the value of conductivity through four-probe resistance sheet measurements. The conductivity was acquired by measuring the resistance and thickness of the film, and the values are shown in Table 3. As a result, it was measured to be 51.0 S/cm and 45.5 S/cm, respectively. The multi-vacancy-defect sample showed lower electrical conductivity and it was attributed to the creation of more defect sites through the removal of the metal oxide NPs

## 4. Conclusions

We successfully formed metal oxide NPs on the MWCNTs and subsequently removed these NPs from the MWCNTs. Although functional groups containing oxygen can be created on the surface of the CNTs to increase their adsorption capacity, they might hinder the diffusion of adsorption molecules or reduce the specific surface area. Herein, we report a method to increase the specific surface area of MWCNTs without reducing the adsorption capacity of the resulting dangling bonds, by forming defects. We employed facile methods, such as preparing a composite without using vacuum or gas and removing metal oxide NPs through simple acid treatment. The formation of various metal oxide NPs was confirmed via TEM observations, and the type of metal oxide and crystal structure of the NPs were determined through XRD analysis. In addition, the removal of NPs increased the specific surface area and decreased the G/D ratio, resulting in defects. The maximum increase in the specific surface area (1273.37 m^2^/g) and the maximum decrease in the G/D ratio (0.74) were observed in the case of Co_3_O_4_. Therefore, more particles were formed while using Co than other types of metals, resulting in more defects.

## Figures and Tables

**Table 1:** BET specific surface areas of purified MWCNTs and multi-vacancy-defect MWCNTs after removal of metal oxide NPs.

	Purified MWCNTs	MWCNTs with Co_3_O_4_ NPs Removed	MWCNTs with Fe_2_O_3_ NPs Removed	MWCNTs with Mn_3_O_4_ NPs Removed	MWCNTs with NiO NPs Removed
BET Specific Surface Area (m^2^/g)	214.01	1273.37	253.52	266.12	218.18
Difference in BET Specific Surface Area (m^2^/g)		1059.36	39.51	52.11	4.17

**Table 2:** G/D ratio value.

Type	G/D Ratio
Purified MWCNTs	0.99
MWCNTs with Co_3_O_4_ NPs removed	0.74
MWCNTs with Fe_2_O_3_ NPs removed	0.90
MWCNTs with Mn_3_O_4_ NPs removed	0.89
MWCNTs with NiO NPs removed	0.92

**Table 3:** Electrical conductivity value.

	Purified MWCNTs	Multi-Vacancy-Defect MWCNTs
Thickness of film (μm)	14	10
Resistance (Ω)	14	22
Conductivity (S/cm)	51.0	45.5

## Data Availability

Not applicable.
